# Bihar’s Pioneering School-Based Deworming Programme: Lessons Learned in Deworming over 17 Million Indian School-Age Children in One Sustainable Campaign

**DOI:** 10.1371/journal.pntd.0004106

**Published:** 2015-11-19

**Authors:** Lesley J. Drake, Sarman Singh, C. K. Mishra, Amarjeet Sinha, Sanjay Kumar, Rajesh Bhushan, T. Deirdre Hollingsworth, Laura J. Appleby, Rakesh Kumar, Kriti Sharma, Yogita Kumar, Sri Raman, Stalin Chakrabarty, Jimmy H. Kihara, N. K. Gunawardena, Grace Hollister, Vandana Kumar, Anish Ankur, Babul Prasad, Sushma Ramachandran, Alissa Fishbane, Prerna Makkar

**Affiliations:** 1 Partnership for Child Development, Imperial College London, School of Public Health, Faculty of Medicine, Imperial College London, London, United Kingdom; 2 Deworm the World, Washington, D.C., United States of America; 3 London Centre for Neglected Tropical Disease Research, Imperial College London, School of Public Health, Faculty of Medicine, London, United Kingdom; 4 Division of Clinical Microbiology & Molecular Medicine, All India Institute of Medical Sciences, New Delhi, India; 5 Department of Health and Family Welfare, Government of Bihar, Bihar, India; 6 State Health Society, Bihar, India; 7 Bihar Education Project Council, Bihar, India; 8 School of Life Sciences, University of Warwick, Coventry, United Kingdom; 9 Warwick Mathematics Institute, University of Warwick, Coventry, United Kingdom; 10 Department of Clinical Sciences, Liverpool School of Tropical Medicine, Liverpool, United Kingdom; 11 Eastern and Southern Africa Centre of International Parasite Control, Kenya Medical Research Institute (KEMRI), Nairobi, Kenya; 12 Department of Parasitology, University of Kelaniya, Ragama, Sri Lanka; 13 Innovations for Poverty Action, New Haven, Connecticut, United States of America; 14 Evidence Action, Washington, D.C., United States of America; Case Western Reserve University School of Medicine, UNITED STATES

## Introduction

Globally, more than 600 million school-age children are at risk of infection with soil-transmitted helminths (STH) and require treatment [1]. These infected children frequently carry the largest burden of disease in a community and are at greater risk of malnutrition and anaemia [2–5], with detrimental effects on educational access and learning as well as mental and physical performance [4,6–10]. Many of these detrimental effects of helminth infection, however, are reversible with antihelminthic drugs [9–11]; thus, the World Health Organization (WHO) advocates reaching a minimum target of regular administration of antihelminthics to at least 75%, and up to 100%, of school-age children at risk of morbidity from STH infection by 2020 [1,12].

The high levels of safety and efficacy of antihelminthic tablets make them ideal for mass drug administration (MDA), and recommended control efforts consist of antihelminthic treatment, administered through MDA, once a year for school-age children in whom STH infection prevalence is between 20% and 49% and twice a year for all school-age children in whom prevalence is at least 50% [13]. By providing easy access to large numbers of children in a structured setting, the school-based deworming model has been successfully used to administer these antihelminthics in multiple settings [14–16]. Thus, school-based deworming, in which the point of care for children is the school and the teachers are administers of the drugs, with critical oversight by health care staff, is recommended in order to cost-effectively and efficiently reach large numbers of children [14–17]. Despite the availability of cheap and efficacious drugs, WHO goals of reaching 75% of at-risk children by 2020 is not on target [18]. Clearly, in order to achieve WHO goals of reaching 75% by 2020, an effort needs to be taken to increase the scale and coverage of deworming programmes to regional and national levels. Box 1 highlights the key elements of a deworming programme implemented in Bihar, which presents a scalable and sustainable school-based model, using the tools available and harnessing existing structures to create a successful, structured deworming programme.

**Box 1. Key Elements for Success of the Deworming Programme in Bihar**

1. **Institutional Framework**
  ◆ Ownership of the programme by the government led to increased programme sustainability and continued financial and governmental support.
  ◆ Additionally, the existence of a multi-sectoral coordination committee (the State School Health Coordination Committee [SSHCC]) provided the necessary supervision, flexibility, and direction to address challenges arising during the programme while ensuring the sustained high profile of the programme.
2. **Evidence-Based Design and Implementation**
  ◆ The catalytic role of development partners helped build a strong, evidence-based programme and resulted in overall reduced costs as well as additional high-level government support.
3. **Collaboration, Communication, and Coordination**
  ◆ Throughout the design and implementation of the programme, close communication and collaboration among different state government bodies, in particular the health and education sectors, enabled the leveraging of their respective physical infrastructures and human resources and maximized coverage while reducing the overall cost of the programme.
  ◆ High levels of acceptability of the deworming programme by the community was a result of context-relevant community awareness campaigns that built local ownership of the programme, prevented a purely “top-down” approach to the programme, and increased participation on the deworming day.

India is estimated to account for more than a quarter of all children requiring treatment for STH globally [1,19], and in 2009 the State Government of Bihar, in the eastern region of India, planned to initiate a state-wide, evidence-based deworming programme run out of state-schools and with technical support from Deworm the World (DtW). From inception, DtW was technically and administratively supported by Partnership for Child Development (PCD) and Innovations for Poverty Action (IPA) and led by PCD’s Executive Director Dr Lesley Drake, who was seconded to DtW to lead the campaign. This joint initiative is reflected in this paper with the term DtW/PCD.

Bihar is one of India’s poorest states, with 8.5% of India’s population and only 1.6% of its gross domestic product (GDP) [20]. The population of Bihar was estimated to be over 104 million in the 2011 census [21] with approximately 28% between 6–14 years of age [21]. Surveys conducted in 2010–2011 found a high prevalence of STH throughout Bihar state [22], and subsequent predictive mapping indicated that at least annual, and in some cases biannual, MDA treatment would be required [17].

This paper outlines the political environment, development, and implementation of all stages of this pioneering and sustainable large-scale deworming programme, which led to the treatment of over 17 million school-age children in Bihar state. In addition, the synergistic support provided by all partners ensured a rapid rollout of the programme, which went from conception to implementation to treatment in less than 12 months, and deworming was rolled out across the state over three months.

## Institutional Framework

Two flagship programmes of the central government, the National Rural Health Mission (NRHM) and the Indian national educational program Sarva Shiksha Abhiyan (SSA) (or Universalization of Elementary Education) ensured that sufficient operational funding was available within the state government to scale up feasible programmes in the areas of health and education. Thus, in 2009, with financial backing from these institutions and endorsement by high-ranking politicians and bureaucrats willing to support evidence-based, cost-effective solutions in health and education, the school-based deworming MDA programme in Bihar was initiated.

**Fig 1:**
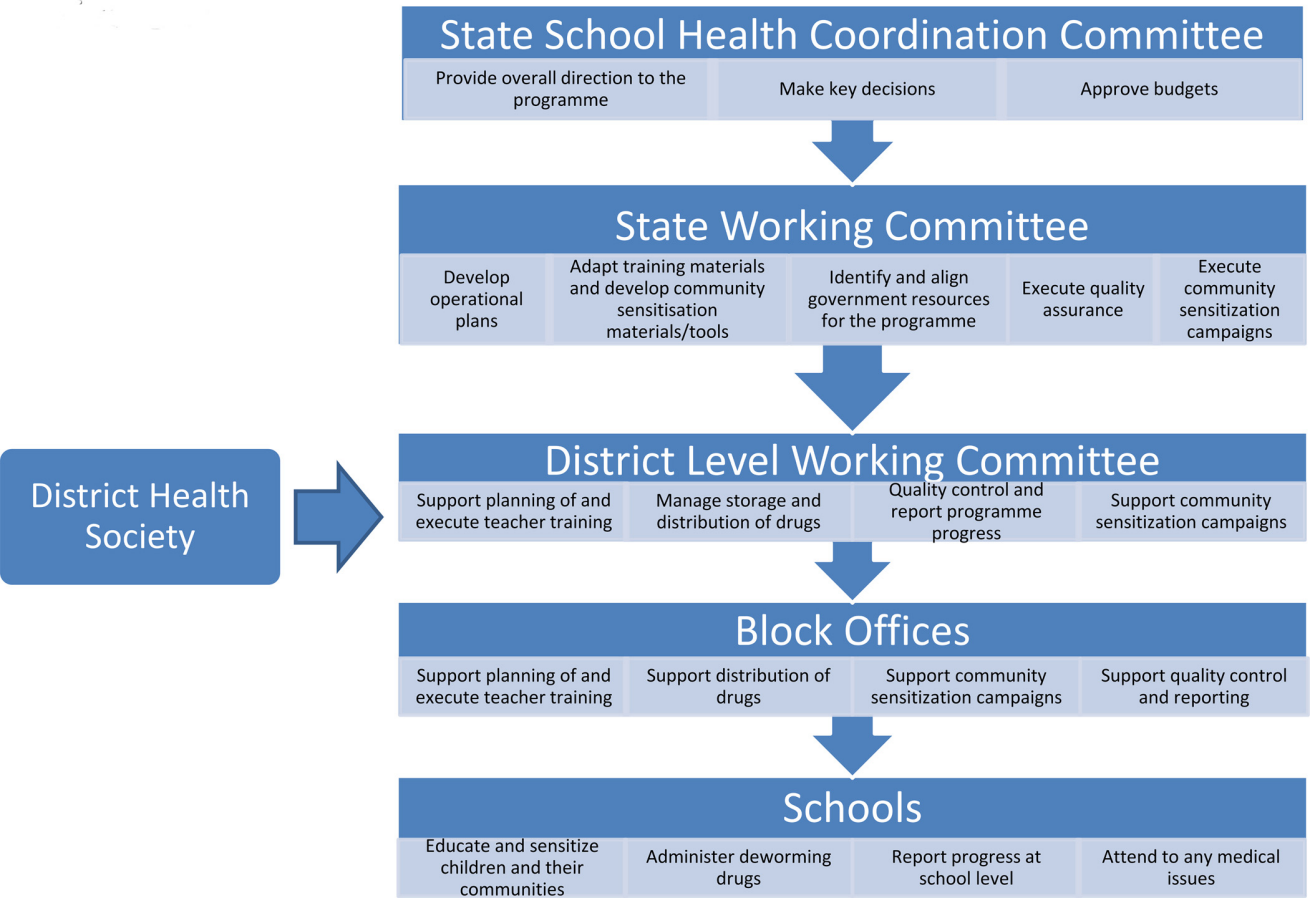
Structure of the state government ministries and offices and respective roles in the deworming programme in Bihar state. Leveraging of the existing state government structures was critical for the successful rollout of large-scale operations, including drug procurement and delivery, training, community sensitization, and reporting.

The development partners (DtW/PCD) provided the necessary high-level advocacy for support by high-ranking bureaucrats, which led to a Memorandum of Understanding among key stakeholders, including the state departments of health and education. In turn, this partnership led to the establishment of a steering committee called the State School Health Coordination Committee (SSHCC), which served as the main decision-making and governance structure for the programme and was ultimately critical for the overall success of the programme in Bihar. The steering committee was composed of representatives from the State Health Society Bihar (SHSB) (part of the Ministry of Health & Family Welfare ([MoHFW]) and the Bihar Education Project Council (BEPC) (part of the Ministry of Human Resource Development [MoHRD]), as well as DtW/PCD. The SSHCC was responsible for driving convergence between both the health and education state departments, from state level to village level, and provided direction, supervision, and timely approvals to the programme. A clear structure for all government and community level players lent the necessary support and sustainability at all levels of the deworming programme, as illustrated in Fig 1.

## Evidence-Based Design

The evidence base for the programme was financed, coordinated, and conducted by DtW/PCD and the All India Institute of Medical Sciences (AIIMS), New Delhi. In the absence of any previous data collection on the prevalence or intensity of STH in Bihar, school-based surveys were conducted to determine baseline prevalence levels and geographical distribution of STH in the state. The surveys, largely conducted according to WHO recommendations for epidemiological data collection [13], were to asses the overall prevalence and intensity of infections, so as to guide the programme design concerning treatment strategy and scale. Surveys were conducted across six diverse districts—Patna, Supaul, Gopalganj, Araria, Muzaffarpur, and Aurangabad—selected based on diversity in land surface temperatures, socioeconomic profiles, and climatic, sanitation, and environmental indicators. For logistical and practical reasons, the surveys were carried out in two phases; the first phase was conducted in May–June 2010 across two districts covering 40 schools and a total of 2,079 school-age children, and the second phase was conducted in January–February 2011, covering 20 schools and 1,159 school-age children. A total of 3,238 school-age children in 60 schools were surveyed.

Stool samples were collected from school children, processed, and examined for eggs of STH (*Ascaris*, *Trichuris*, and hookworm) at district-level laboratories, state-level laboratories, or at the AIIMS laboratory based in New Delhi, depending on infrastructure, logistical ease, and facilities. Two techniques, Kato-Katz [23] and Mini Parasep SF (Apacor, Berkshire, United Kingdom), were used to determine presence or absence of helminth infection and hence prevalence of STH in the regions (see Box 2 and Smith, J. L., Koukounari, A., Kihara, J. H., Gunawardena, N. K., Kumar, S., Makkar, P., Drake, L., Singh, S., and Dixon, R: Evaluation of Parasep SF and Kato-Katz as diagnostic techniques for soil-transmitted helminth surveys in Bihar State, India: performance and treatment recommendations, Submitted). The use of two diagnostic techniques provided flexibility to a large scale programme, ensuring data collection was as rapid and accurate as possible considering technical, logistical, and cultural constraints.

**Box 2. The Necessity of Appropriate Parasitological Tests for Survey Setting**

In certain districts, logistical reasons such as limited availability of local laboratory facilities and time constraints precluded the use of Kato-Katz; thus, Mini Parasep SF was used, in agreement with the state government, as an alternative technique to analyse approximately one-third of all the samples. Despite Mini Parasep SF techniques being found to have overall less sensitivity than Kato-Katz ([24] and Smith JL, Koukounari A, Kihara JH, Gunawardena NK, Kumar S, Makkar P, Drake L, Singh S, and Dixon R: Evaluation of Parasep SF and Kato-Katz as diagnostic techniques for soil-transmitted helminth surveys in Bihar State, India: performance and treatment recommendations [Submitted]) because of high prevalence of STH in the area, the sensitivity of the Mini Parasep SF still ensured annual MDA of children in all surveyed districts where prevalence was over 20% [1]. Furthermore, its use was deemed preferable to the Kato-Katz in certain districts, as logistical constraints, such as limited trained personnel and laboratory facilities in rural areas, meant that samples could not be processed within the recommended time frame for accurate egg count using Kato-Katz techniques [25]. Additionally, the use of the Mini Parasep SF decreased the overall costs of the programme by centralizing some of the more technical procedures, and it was frequently considered a more acceptable tool amongst the technicians in certain districts because of minimal handling of stool samples.

Given the vast scale of the proposed programme as well as the rural and hard-to-access schools, it was necessary to find practical measures for conducting work that could be sustainable. Here, for purposes of determining programme scale and coverage, the Mini Parasep SF provided an alternative method that was fast, easy, and culturally acceptable, without the same requirements as the Kato-Katz for extensive labour input and a local, well-equipped laboratory at the point of sample collection. Furthermore, the use of the Mini Parasep SF provided an adequate measure of STH prevalence and demonstrated that for rapid scale-up of a large-scale deworming programme, compromising on minor differences in sensitivity of detection does not necessarily compromise on eventual programmatic coverage, a factor for consideration in achieving scale for deworming programmes globally. Importantly, the successful use of two different diagnostic techniques illustrates the benefits of maintaining flexibility in large scale programmes in which techniques may need to be adapted according to the prevailing programme conditions.

The overall prevalence of STH infection from over 3,000 school-age children across 60 schools in the Bihar was found to be 42%, ranging from 10% up to 96% across surveyed schools and from 23% up to 80% in surveyed districts. All surveyed districts exceeded the WHO-defined prevalence threshold for annual MDA, and four of the six surveyed districts exceeded the threshold for biannual MDA [13]. Further details on the survey sites as well as final prevalence data on STH infection can be found in the paper by Greenland et al 2015 [22].

**Fig 2:**
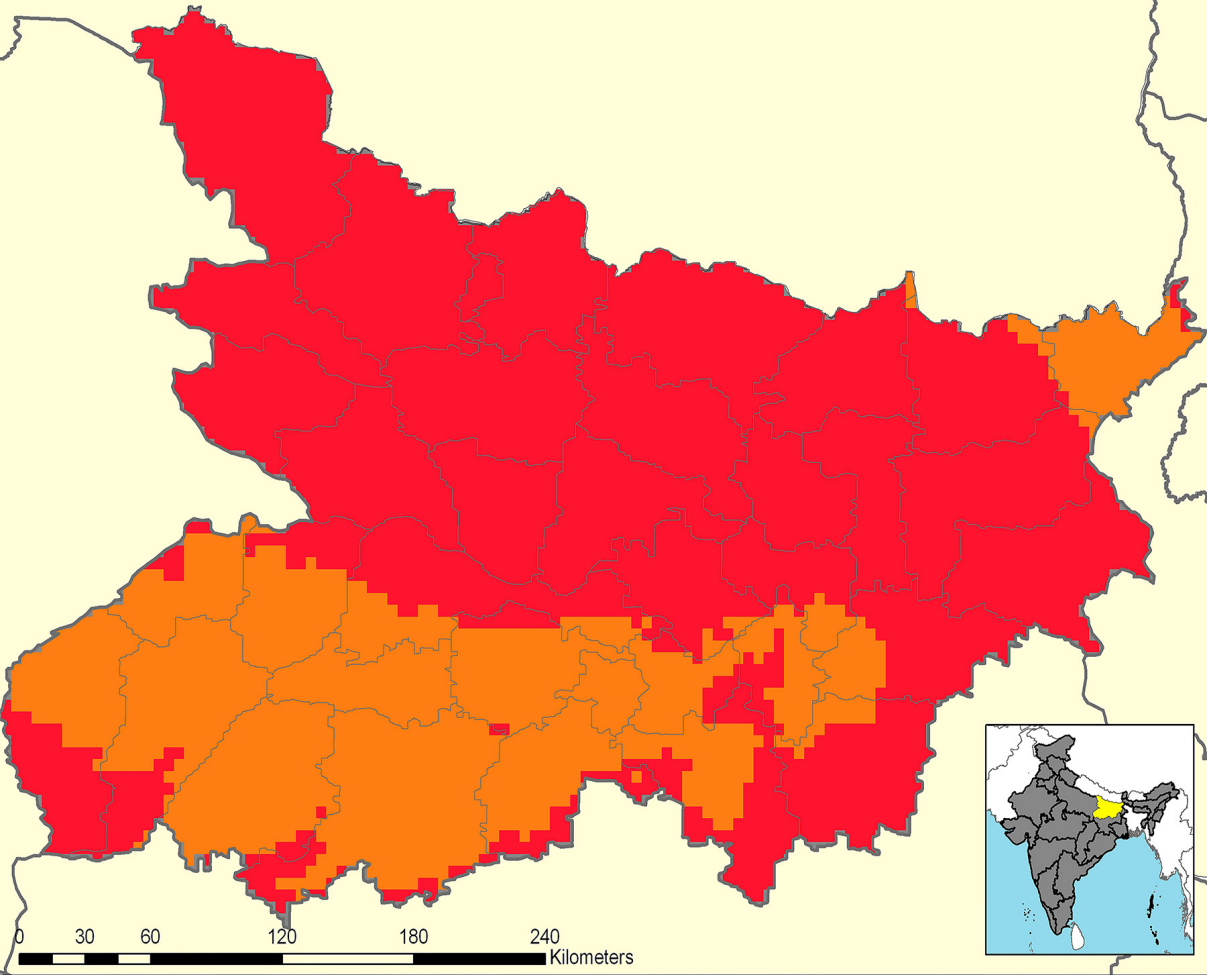
Predictive prevalence map of soil-transmitted helminths in Bihar state. Predictive mapping was used to inform the state wide deworming coverage in Bihar. The map shows areas of high prevalence (≥50% infection) in red, and moderate prevalence (≥20% and <50% infection) in orange. The map presented was developed by Jenny Smith as part of the Global Atlas of Helminth Infections project (www.thiswormyworld.org)

The worm prevalence results were extrapolated to all districts across the state to generate the predictive prevalence map, shown in Fig 2, using a model which included climatic and socioeconomic data [26]. This map was an essential tool in designing the deworming treatment programme for Bihar state. Fig 2 illustrates the high predicted prevalence of STH infection within Bihar state, with the majority of districts estimated to have over 50% STH prevalence and thus warranting biannual treatment according to current WHO thresholds [13].

## Training Programme

**Fig 3:**
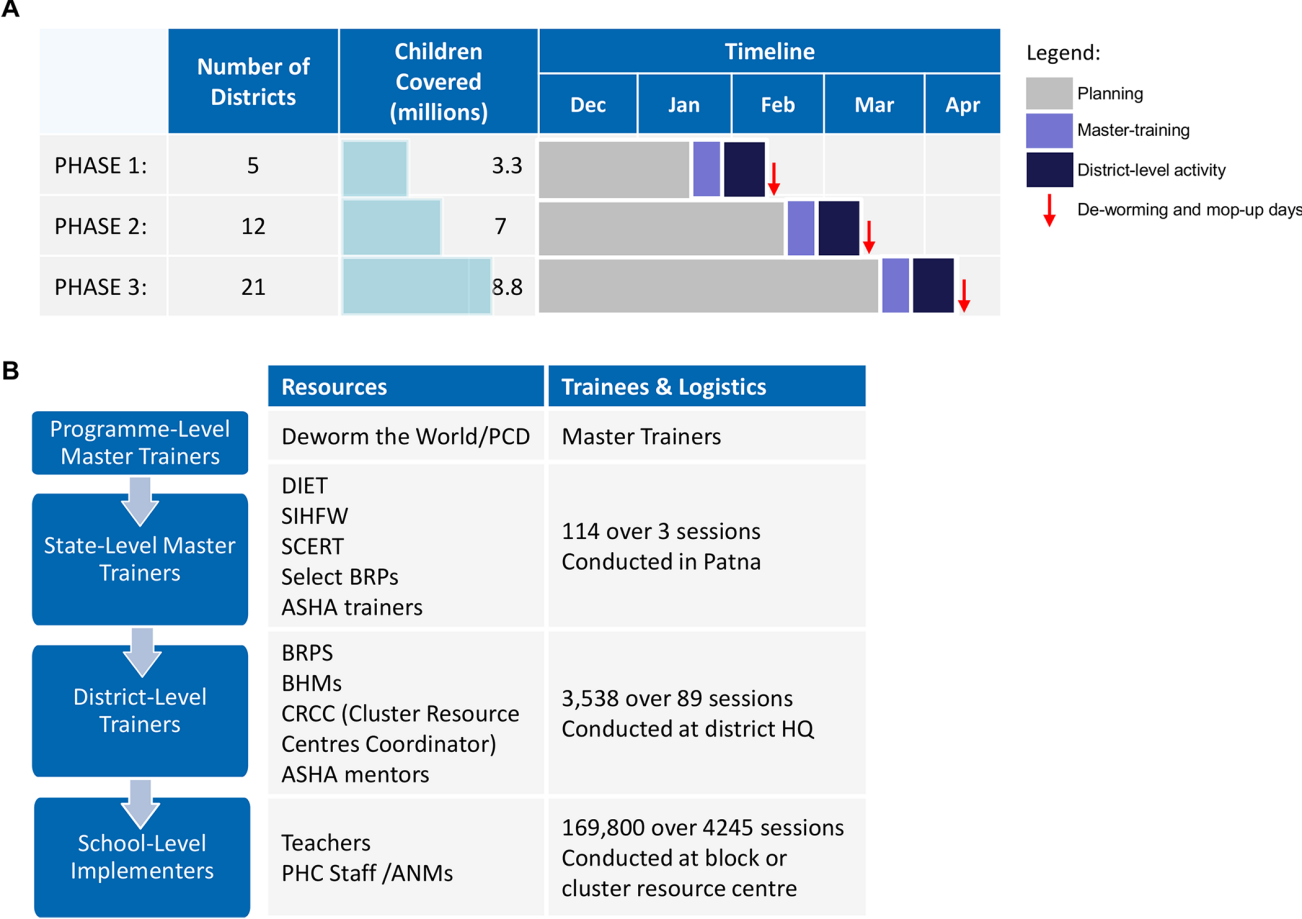
School-based deworming cascaded training and programme rollout in Bihar. **A,** The incremental three-phased approach, including numbers targeted in each phase. Timelines are shown, with indications of planning phases (light grey), training phases (light purple), and district-level activity (dark blue). Deworming days are indicated in red arrows. District-level activity includes school-level activities and the cascaded training of teachers and Primary Health Centre staff. **B**, Structure and design of cascaded training sessions from programme level to school level, including numbers trained and trainees. The cascaded system of training and dissemination of materials led to significant cost reductions and maximized use of available resources. Abbreviations: DtW/PCD, Deworm the World and the Partnership for Child Development; DIET, District Institute of Education and Training; SIHFW, State Institute of Health and Family Welfare; SCERT, State Council of Education Research and Training; CRP, Cluster Resource Persons; ASHA, Accredited Social Health Activist; BRP, Block Resource Persons; BHM, block health managers; CRCC, Cluster Resource Centres Coordinator; PHC, Primary Health Centre; ANM, Auxiliary Nurse Midwife; HQ, headquarters.

Nearly 140,000 teachers and 20,000 healthcare staff throughout Bihar were trained to deliver the deworming tablets, monitor the programme, record and handle adverse effects, and help build awareness within the community. A cascaded training system was used to train this number of individuals in a short period of time. Training was rolled out in three phases to coordinate with the deworming and “mop-up” days being conducted across the state. Fig 3A illustrates the timeline of the rollout of planning, training, and deworming activities. For each phase, state-level master training took place over two days, district-level training over one day, and school-level training over half a day. District and school-level training took place after the state-level master training and the week before the school-based deworming and “mop-up” days. The structure of the cascaded training plan is shown in Fig 3B. The cascaded training model consisted of teachers and healthcare staff who attended centralized, state-level training and passed on training knowledge to teachers at the district and subdistrict levels, thereby maximizing resource capacity and increasing the speed of dissemination. Furthermore, such a cascaded training design provided the opportunity for training materials, reporting and monitoring forms, and deworming drugs to be easily and cost-effectively distributed throughout the state.

## Community Awareness and Participation

**Fig 4:**
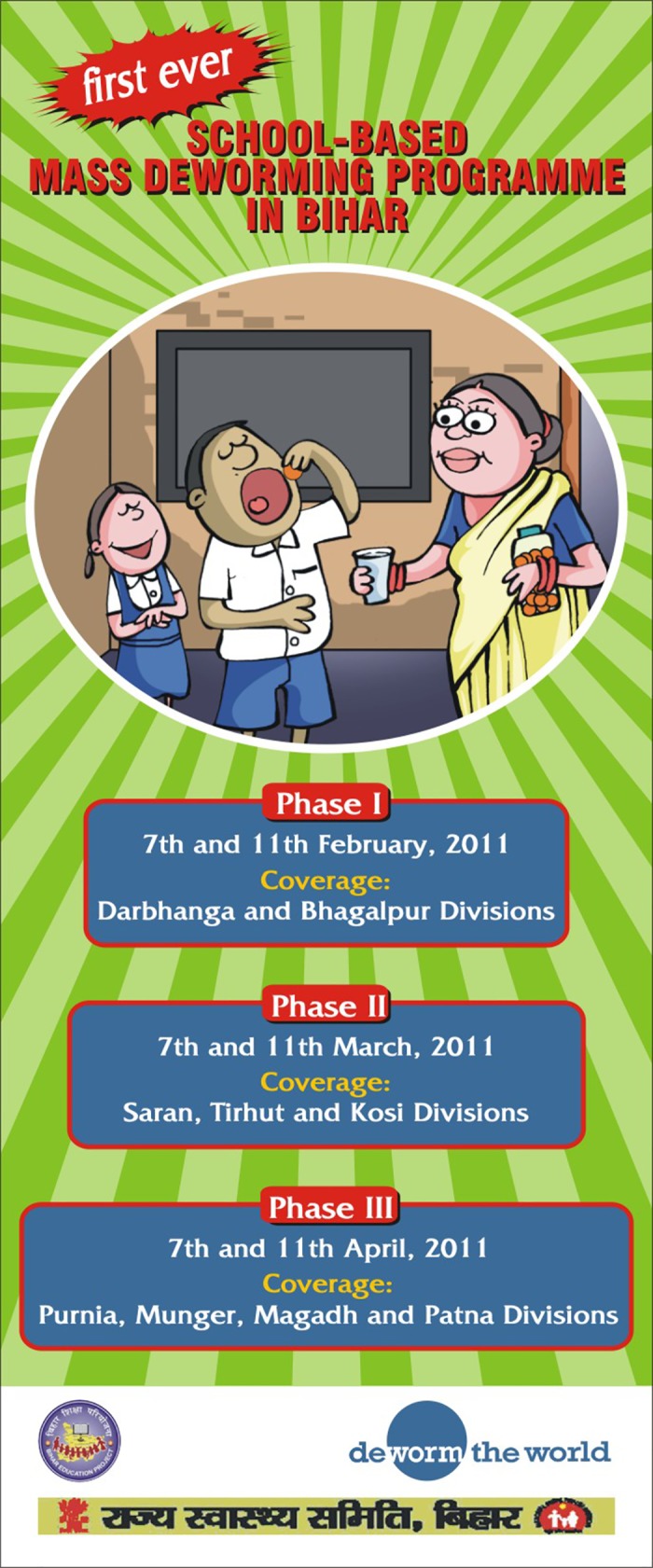
Examples of community sensitization and awareness posters. Phase-wise information on the programme. The posters were also translated into Hindi and demonstrate the level of dissemination of programme information to the communities, including dates to expect the deworming day to occur as well as the”mop-up” days to cover children who could not attend the deworming day. The repeating dates across the months provided a tactic with which to galvanize the deworming days in community members’ minds.

In order to sufficiently sensitize and educate the community about the deworming programme, both in terms of why the government was initiating it as well as the outcomes and potential side effects, materials specific to India and the context of Bihar were developed in the local language (Hindi). Key government partners SHSB and BEPC and Bihar’s Public Relations Department (PRD), together with DtW/PCD, were responsible for developing and dissemination of the press and community sensitization materials. Fig 4 presents an example of a poster that was produced for this purpose. In the weeks leading up to school-based deworming, the programme was communicated throughout the community, particularly to children, parents, teachers, community leaders, and local officials. Communication strategies included newspaper appeals by the government to the public, radio jingles, street plays, school plays, and *prabhat pheris* (morning processions of children through their neighborhoods shouting deworming slogans). In addition there was extensive press coverage, including radio broadcasts, press conferences, and media interviews of the various government officials from both the Ministry of Education and Ministry of Health. Wherever possible, the message of deworming was associated with a “Right to Education” (RTE) message, as part of an extensive state campaign. This linking of the two campaigns leveraged additional resources for sensitization and ensured sustainability and acceptability of the programme because of the continuation of an existing message of RTE, as well as preexisting local involvement in the messaging.

The communication strategy provided the opportunity to disseminate specific information, such as details on the safety of deworming and expected adverse effects, and procedures to follow if adverse effects were experienced. In addition, programme dates and details for procedures for non-enrolled children who wished to receive treatment were included in public broadcasting messages. Involving the community encouraged greater participation on the initial designated deworming day and subsequent”mop-up” day and allowed inclusion of younger siblings and non-enrolled children, maximizing programme coverage. There was only one adverse event to albendazole treatment reported in Bihar. The case was addressed swiftly and appropriately by administering teachers and medical authorities. Because of strong local involvement and community sensitization efforts leading up to the programme, the school and community did not overreact to this event, thereby demonstrating the importance of both communication and clear, responsive protocols for adverse events.

## Deworming Days

Dates for deworming days were advertised across the state. These deworming days were rolled out in three phases between February and April of 2011 (Fig 4), targeting over 21 million school-age children and reaching over 17 million children both enrolled and non-enrolled. As the programme predated the global drug donation of albendazole and mebendazole [27], enough low-cost, quality-assured drugs were procured to treat the estimated 21 million school-age children in Bihar, plus an additional 20% added to this number of tablets in order to treat out-of-school and pre-school children who were encouraged to attend deworming and “mop-up” days.

Of the 17 million children treated in Bihar, 1 million were not currently enrolled in school. In addition, teachers were offered tablets for both themselves and their family members, which served as an incentive and contributed to partial coverage of the community, a necessary measure in the goal to eliminate STH as a public health problem [28].

Drugs were procured by the SHSB from the generic drugs manufacturer Omega Biotech Limited (Uttarakhand, India). The drug manufacturer, selected from a government preapproved vendor list, was subject to a site visit and quality control was undertaken on a sample of the albendazole tablets at an independent laboratory. Chewable albendazole 400 mg tablets were administered to each child at the time of deworming as per WHO guidelines [13].

Deworming was conducted at each of the over 67,000 schools in the state by two trained teachers per school. Schoolchildren from 6–14 years old were dewormed; in addition, out-of-school children and pre-school children were encouraged to attend school to receive deworming medication. Children who had not eaten prior to the dispensation of the deworming tablets were provided with biscuits to minimize adverse effects of the drugs [29]. A source of clean water was already available or made available at every school for the child to take with the chewed tablet if needed.

## Opportunities for Integration

At the same time as baseline surveys were being conducted, India was due to commence lymphatic filariasis (LF) treatment within Bihar state as part of the annual National Filaria Control Programme (NFCP). The control strategy consists of coadministration of diethylcarbamazine citrate (DEC) with albendazole. The administration of albendazole, one of the two drugs which are used for treating STH, could have resulted in duplication of efforts if the school-based deworming programme was not coordinated with the NFCP. Thus, because of the high prevalence of STH infections in Bihar (Fig 2) and the WHO-recommended treatment strategy of biannual treatment in areas where prevalence is ≥50% [13], the SSHCC in Bihar endeavored to maximize the impact of the existing NFCP programme and ensure at least annual deworming to all at-risk children by implementing the school-based deworming programme at a six-month staggered interval with the NFCP programme.

Coordinating with other neglected tropical disease (NTD) control programmes has been shown to be safe and efficient [30], and in the current climate of NTD control and elimination, integration amongst different NTD control programmes is recommended wherever feasible and applicable [17,31,32]. The deworming programme presented in this paper demonstrates that control programme activities can be coordinated effectively and simply, ensuring maximum chemotherapy coverage for multiple infections with the potential for greater impact on NTDs.

## Monitoring and Evaluation

Monitoring and evaluation was conducted at every level of training and programme implementation in Bihar. Side effects were monitored and recorded, and an adverse event protocol developed by SHSB had been distributed prior to the deworming days to all health facilities to advise on appropriate management. Any adverse effects were to be treated by the health department of Bihar.

The children were monitored by the teachers at the time of administering the deworming drugs to ensure that tablets were chewed and ingested, and a record was made of successful treatment for each child. Health workers served as independent monitors to ensure that distribution and recording of treatment was being conducted correctly. Children who had missed deworming day were targeted for treatment during a”mop-up” phase conducted a few days after the main deworming day. External monitors and auditors were deployed around Bihar to provide independent assessment on the programme success. These monitors and auditors covered approximately 5% of the schools in Bihar to check coverage and process, including accuracy of treatment records by teachers, proper administration and monitoring of tablet distribution, as well as accuracy and thoroughness of educating children on worms. In addition, individual, school, cluster, block, and district-level information on numbers of tablets distributed were all recorded and cross-checked for inaccuracies in reporting.

## Leveraging Finances through Collaboration

The strong evidence base that had been developed as part of the programme bolstered high-level advocacy and support for the programme. Indeed, a preliminary analysis of cost sharing between development partners and the state government suggested that support provided by the development partners catalyzed investment of at least three times the initial investment value in additional operational financing from the government—US$500,000 invested by DtW/PCD (with funding from The World Bank and Global Network for Neglected Tropical Diseases) and at least US$1.56 million (calculated on a best-effort basis) invested by the Government of Bihar.

The operational financing and programme implementation from the Government of Bihar was provided through the SHSB, BEPC, and PRD. This additional support was utilized for the procurement of deworming drugs, training of teachers and health staff, community sensitization, and programme publicity. The direct investment, in addition to the high imputed costs of leveraging existing government-funded physical infrastructure and human resources, resulted in even higher levels of resource efficiency and helped further lower programme costs. Thus, the initial technical and financial assistance provided by the development partners had a significant catalytic effect in leveraging larger investment multiples from the state government, contributing to the overall success of the programme.

## Key Outcomes

In 2011, Bihar state successfully implemented its first ever state-wide deworming programme. Treating over 17 million of the 21 million targeted school-age children in over 67,000 schools across Bihar and within a year of programme conceptualization, the programme constitutes the largest school-based deworming exercise ever completed globally. Rolled out across the state in just over three months, the programme reached over 80% coverage, and exceeded WHO recommended targets [13,27].

Funding for the programme was provided by both partners and the state government, with initial investments by partners leading to leveraging of much greater values in direct and imputed investment by the Government of Bihar, specifically by the SHSB, BEPC, and PRD. The SSHCC that was founded as part of the initialization of the programme is still in existence and continues to oversee and coordinate the annual deworming days throughout Bihar. Subsequent rounds of the deworming programme reached 16.3 million children in 2012 and 16.3 million children in 2014, with the numbers reached in the recent round of deworming conducted in February 2015 to be determined. The albendazole tablets used in the programme, initially procured by SHSB in 2011, are now being received from WHO through the recent global drug donation [27], an adaptation that further illustrates the flexibility afforded by the multi-sectoral decision making body SSHCC.

## Way Forward

Large-scale, evidence-based programmes operating from a school-based platform have been shown to be an efficient way to reach large numbers of school-age children with safe and effective drugs, increasing programme impact [15,33,34]. Leveraging teachers as a human resource in addition to healthcare staff provides a cost-effective method for programme delivery. In efforts to control STH infections and reach WHO targets of treating 75% of school-age children by 2020 [12,35], widespread coverage of deworming programmes in different settings will be required.

We have outlined here the implementation by the Bihar state government of a pioneering, large-scale, school-based deworming programme, demonstrating the feasibility of treating millions of children within a few months and with minimal resources. Further mainstreaming into education sector plans would bring costs, both direct and indirect, down even further, providing additional value to these school-based programmes. In addition, using the schools as a platform led to deworming treatment being received by a significant number of out-of-school children who had easy access to schools within their communities. Together, these demonstrate the efficiencies of this platform and the opportunities they provide to reach a large number of children effectively.

The combination of an evidence-based and school-based design, strong political support at all levels, and catalytic technical assistance leveraging additional operational financing from the government led to a deworming programme that was comprehensive, successful, and sustainable in its design.

In India, there are over 250 million children between the ages of 5 and 14, with a 97% school enrollment rate [21,36]. The deworming programme run in Bihar state predates the global drug donation announced at the London Declaration [27], yet it still constitutes the largest school-based deworming programme ever implemented. With the advent of the donation and the proof of concept shown in Bihar, together with continuing support from government initiatives such as NRHM and SSA, this model is now being rolled out across the country as part of MoHFW’s recently announced National Deworming Day. The first round of deworming was conducted in February 2015 and targeted over 140 million children across 11 states in India, which will lead to improvements in education, health, and productivity for millions of school-age children across the country.
